# Craniofacial morphology and its association with masseter and medial pterygoid muscle thickness among adults from a population-based sample

**DOI:** 10.1007/s00784-026-07102-4

**Published:** 2026-08-25

**Authors:** Annalena Spilz, Christian Schwahn, Karl-Friedrich Krey, Robin Bülow, Henry Völzke, Markus Krüger, Amro Daboul

**Affiliations:** 1 Department of Prosthetic Dentistry, Gerodontology, and Biomaterials, University Medicine Greifswald, Fleischmannstraße 42-44, Greifswald, 17475 Germany; 2 Department of Orthodontics, University Medicine Greifswald, Fleischmannstraße 42-44, Greifswald, 17475 Germany; 3 Institute of Diagnostic Radiology and Neuroradiology, University Medicine Greifswald, Walther-Rathenau-Straße 46, Greifswald, 17475 Germany; 4 Institute for Community Medicine, University Medicine Greifswald, Walther Rathenau Straße 48, Greifswald, 17475 Germany; 5 Zentrum für Zahn-, Mund- und Kieferheilkunde, Walther- Rathenau-Straße 42a, Greifswald, 17475 Germany

**Keywords:** Masticatory muscles, Craniofacial morphology, Geometric morphometry

## Abstract

**Objectives:**

The association between craniofacial morphology and the cross-sectional areas (CSA) of the masseter and medial pterygoid muscles using Procrustes-based geometric morphometrics on magnetic resonance imaging (MRI) data was investigated.

**Materials and methods:**

This cross-sectional study included 692 participants from the first examination wave of the second cohort of the population-based Study of Health in Pomerania (SHIP-TREND-0). Craniofacial landmarks were identified on MRI scans, and muscle CSAs were measured using image segmentation techniques. Principal component (PC) analysis was used to assess morphological variation. Regression models adjusted for sex, age, education, dental status, body height and weight, and centroid size were used to estimate associations between craniofacial shape and muscle CSA.

**Results:**

PC1, which primarily captured facial height and cranial base length, and PC2, which represented maxillary width and facial prominence, were associated with medial pterygoid muscle CSA. Longer faces (PC1) were associated with thinner pterygoid muscles, while wider midfaces (PC2) were associated with larger CSA (*P* = 0.0038 and *P* = 0.0005, respectively). Partial R² of PC1 through PC7 was 0.038 for pterygoid muscles and 0.016 for masseter muscles (*P* < 0.0001 and *P* = 0.0225 for joint tests, respectively). There was a stronger association between number of teeth and masseter muscles than with pterygoid muscles (partial R² 0.076 and 0.026, respectively).

**Conclusions:**

Craniofacial variation, particularly in terms of facial height and midface width, was associated with the medial pterygoid muscle rather than the masseter muscle.

**Clinical relevance:**

Associations suggest a potential anatomical basis for the study of myofunctional disorders.

**Supplementary Information:**

The online version contains supplementary material available at https://doi.org/10.1007/s00784-026-07102-4.

## Introduction

Craniofacial skeletal development and masticatory muscle growth are closely interconnected, influencing each other throughout life [1]. The formation and remodeling of craniofacial bones are dictated not only by genetic factors but also by mechanical forces exerted by the masticatory muscles. Masticatory activity plays a crucial role in bone adaptation by stimulating growth at sutures and modifying craniofacial skeletal form [2, 3]. Conversely, the size and shape of craniofacial bones provide the framework for muscle attachment, which in turn influences muscle development and activity [4].

Most studies [5–7] on masticatory muscles have relied on indirect representations of muscle force, such as electromyography and bite force tests. A more direct approach involves measuring the cross-sectional area of the muscles, which serves as an indicator of maximum muscle force [8]. Several studies have reported the cross-sectional areas and volumes of the masticatory muscles, exploring their relationship with craniofacial morphology. Some findings suggest a positive association between the cross-sectional areas of the masseter, pterygoid, and temporalis muscles with facial, bizygomatic, and cranial width [9, 10], whereas other studies indicate negative associations with vertical craniofacial dimensions, such as facial and cranial height [11, 12].

Traditional assessments of craniofacial morphology have relied on linear measurements and angular relationships between anatomical landmarks. However, new geometric morphometric tools have been developed to enhance the quantification, analysis, and visualization of morphological variation. These methods use anatomical landmarks on 2D or 3D images, preserving geometric relationships among structures and offering advanced visualization techniques [13, 14].

The aim of this study was to identify the main axes of craniofacial shape variation associated with masseter and medial pterygoid muscle size in a large population-based MRI study, and to examine whether these associations differ between the two muscles after adjustment for relevant confounders.

## Materials and methods

### Study population

The SHIP is a population-based project that currently comprises three independent cohorts. The first, SHIP-START, has conducted four follow-ups since 1997. The second, SHIP-TREND, has conducted one follow-up study since 2008 [15]. The third, SHIP-NEXT, commenced in 2021 with the baseline examination. The SHIP research initiative is a population-based study designed to investigate common risk factors, subclinical disorders, and manifest diseases among adults from northeastern Germany [15].

From 2008 to 2012, 4420 individuals aged 20–79 years participated in SHIP-TREND-0, the baseline examination of the second SHIP cohort, which included a comprehensive medical and dental examination, standardized interviews, and self-administered health-related questionnaires [15]. The entire oral examination was performed by calibrated, licensed dentists, and body height and weight were measured as part of standardized somatometric examinations.

The MRI sequences relevant to this study included T1-weighted head scans with an ultrafast gradient echo sequence (TR 1900 ms, TE 3.37 ms, flip angle 15°, matrix 256 × 176, voxel size 1.0 × 1.0 × 1.0 mm, bandwidth 130 Hz/pixel), and T1-weighted head and neck scans (TR 587 ms, TE 11 ms, flip angle 150°, matrix 256 × 192, voxel size 1.0 × 0.8 × 4.0 mm, bandwidth 150 Hz/pixel). Detailed MRI protocol is described elsewhere [16].

Whole-body MRI was offered as an optional examination to eligible SHIP-TREND participants, and not all participants agreed or were eligible to undergo MRI. MRI was subject to certain exclusion criteria including non-MRI compatible implants, pacemakers or ICD systems, ferromagnetic metal fragments or vascular clips, neurostimulators, medication pumps, or extensive tattoos containing metallic pigments [17]. For the present analysis, MRI data were matched with the corresponding oral examination data, and participants were included only when the relevant head and neck sequences permitted both craniofacial landmark identification and muscle cross-sectional area measurement. MRI images were excluded if they showed artifacts or did not cover the anatomical regions of interest. The final analytical sample comprised 692 participants with suitable MRI images and complete oral and demographic data, which was addressed using inverse probability weighting. The study was approved by the Medical Ethics Committee of the University of Greifswald, and written informed consent was obtained from all participants.

### Craniofacial landmark and muscle measurements

MRI images were analyzed using the open-source DICOM viewer OsiriX v5.8.1 (Pixmeo, Geneva, Switzerland) on two iMac Quad Core i7 workstations equipped with 27-inch monitors (Apple Corp., Cupertino, CA, USA). Four observers participated in a training phase to standardize landmark identification and muscle measurement procedures.

**Fig. 1:**
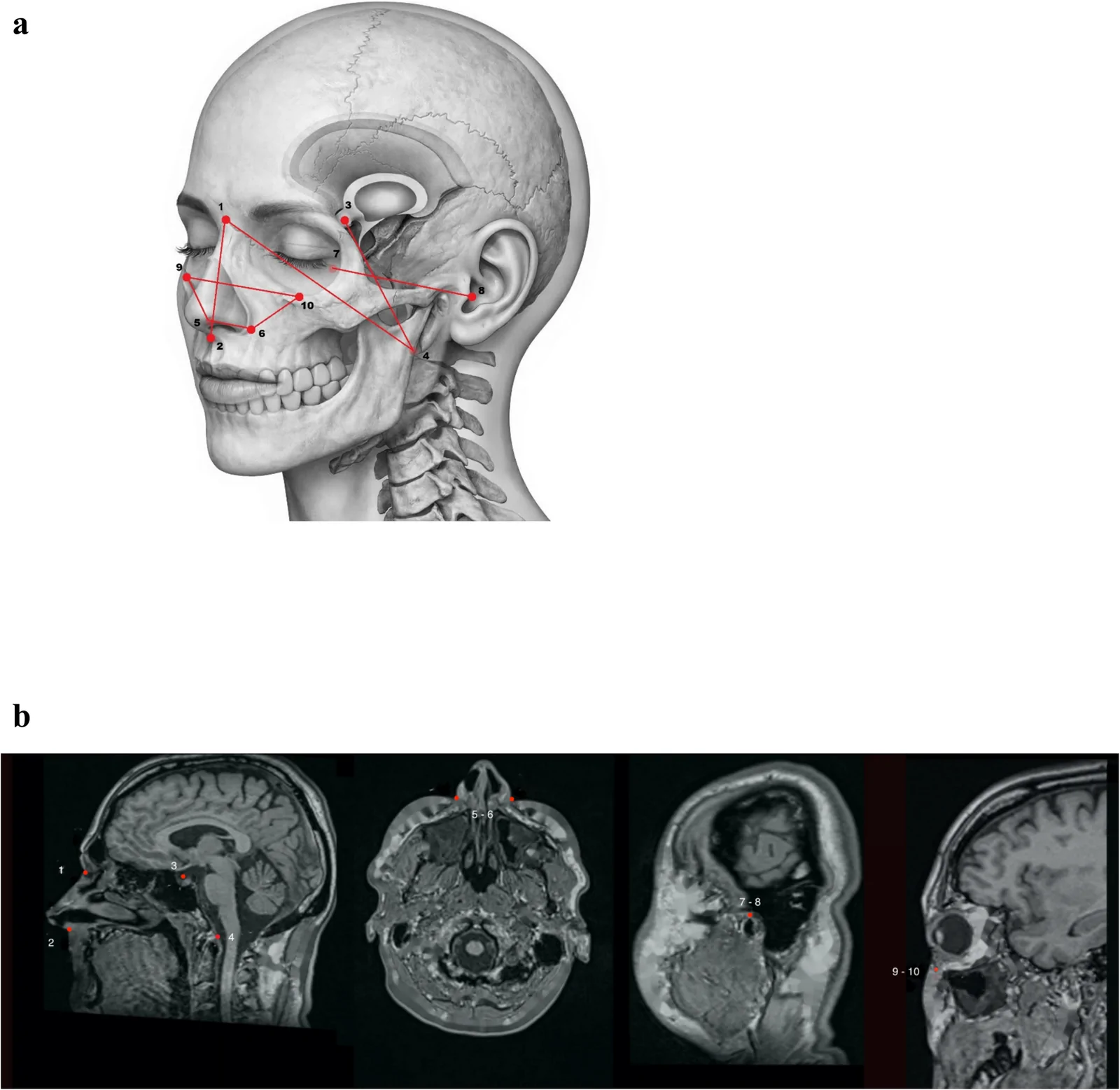
Listing of the craniofacial landmarks used: (1) nasion, (2) subnasale, (3) sella, (4) basion, (5) alare right, (6) alare left, (7) porion right, (8) porion left, (9) orbitale right, (10) orbitale left. Lines indicate the connections used

The craniofacial landmark configuration was selected to capture facial morphological variations in adults and consisted of the following anatomical landmarks (Fig. 1): nasion, subnasale soft, sella, basion, alare right and left, porion right and left, and orbitale right and left. This landmark configuration was selected to capture the main dimensions of craniofacial shape identifiable on MRI, including facial height, width, prominence, and cranial base configuration, reflecting overall craniofacial proportions rather than detailed regional morphology.

The masseter and medial pterygoid muscles were selected because they are closely linked anatomically and functionally as major mandibular elevators. Both muscles could also be assessed in a relatively standardized manner on the available MRI sequences by defining each muscle’s long axis and measuring a perpendicular cross-sectional area. The temporalis was not included because its broad fan-shaped architecture, converging fiber orientation and distinct superficial and deep components made a standardized single cross-sectional measurement less reliable.

**Fig. 2:**
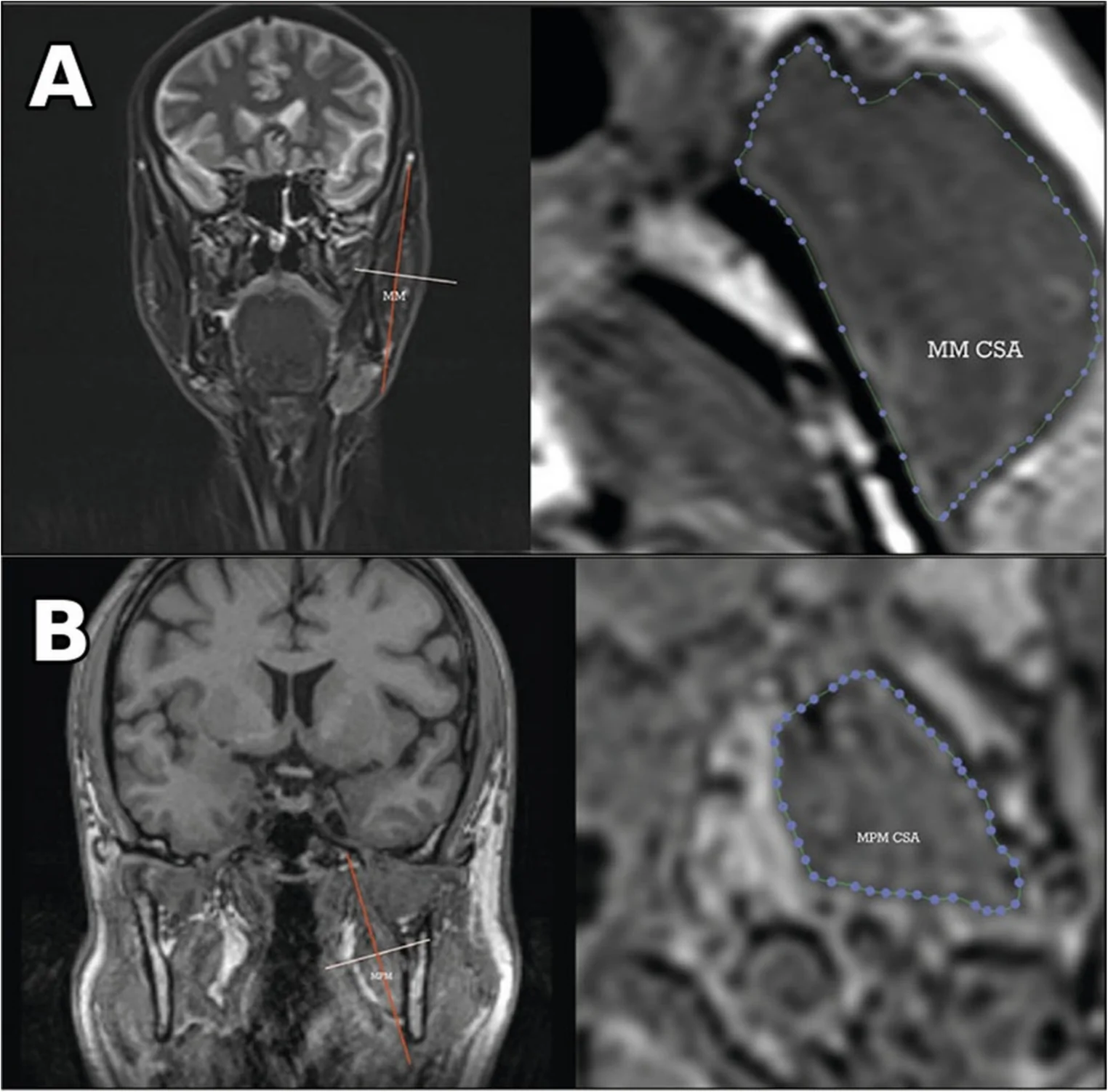
Determination of (**A**) the masseter and (**B**) medial pterygoid muscle’s long axis based on a coronal T2-weighted inversion recovery sequence and assessment of the corresponding cross-sectional area perpendicular to the muscle’s long axis on a transverse T1-weighted sequence

To measure the cross-sectional areas of the masseter and medial pterygoid muscles, the CSA of each muscle was assessed on a plane perpendicular to its long axis. For the masseter, the long axis and medio-lateral inclination were determined separately for each subject on the coronal T2-weighted inversion recovery sequence. For the medial pterygoid, reconstructed multiplanar T1-weighted axial images were used to determine the long axis individually. After defining the long axis, the corresponding CSA was measured at the thickest area on the perpendicular section using segmentation tools in OsiriX. The CSA of each muscle was measured by outlining its borders using the polygon selection tool (Fig. 2). The image was first adjusted to a level of 50, with a window width ranging from 300 to 400, to enable visual discrimination between the muscle and the surrounding connective tissue. The CSAs were measured as the areas inside the polygons in cm². Both left and right muscles were measured separately. Reproducibility of both measurement components (Landmarks and Muscle) was established in previous studies using the same MRI protocols. For muscle CSA, inter- and intra-observer reliability was assessed on 10 image sets before the first measurement session and reassessed on 15 image sets after each subsequent measurement session. The resulting intraclass correlation coefficients (ICCs) ranged from 0.98 to 0.99 for the masseter and from 0.79 to 0.81 for the medial pterygoid [8]. Craniofacial landmark reproducibility was assessed separately, with inter-observer ICCs exceeding 0.90 for all landmark coordinates [18], while the geometric morphometric validation of the landmarks reported centroid size ICCs ranging from 0.81 to 0.98 across the evaluated landmark configurations [19]. These findings demonstrate good reproducibility of both the muscle CSA and craniofacial landmark measurements used in the present study.

### Geometric morphometrics, sampling issues, and statistical analysis

Craniofacial morphology was analyzed using principal component (PC) analysis as a data reduction method to identify the primary PCs of variation in the landmarks. PCs with at least 5% variation were selected using the geomorph package [20, 21] of the R software [22, 23]. The point-based visualization generated from PC analysis were used to visualize morphological characteristics such as facial lengthening or shortening, midface widening, and maxillary orientation along each principal component. To facilitate interpretation, the shape configurations representing the minimum (orange) and maximum (blue) values along each principal component were compared in relation to the muscle CSA. In the upper row (transverse plane), the comparison primarily reflected differences in cranial width; in the second row (frontal plane), changes in cranial length were assessed; and in the lower row (sagittal plane), the focus was on the length of the cranial base and the orientation of the maxilla.

We examined potential selection bias by using recursive partitioning to predict the 692 MRI participants out of the 4420 total SHIP-TREND-0 participants, all of whom were independent observations in this cross-sectional study. This method effectively deals with missing predictor values using surrogate splits [24]. We considered the continuous variables age, teeth, body height, and body weight, as well as sex and school education as predictors. A tree model with a single node for splitting dentate from edentulous participants was created, suggesting the use of inverse probability weights [25, 26] derived from the appropriate tooth count categories [27] (Supplementary Information Table S1). In the main text, we present regression models with these sampling weights, and in the Supplementary Information, we present models without them.

Partial R² values of ordinary least square regression models were used to assess the importance of PCs on masticatory muscle size [24] adjusted for sex, age, the interaction between sex and age, school education, number of teeth (up to 32), body height, body weight, and centroid size. Centroid size is a unit measure of size computed as the square root of the sum of squared distances of all landmarks from the centroid [28]. To address the most important mathematical assumption in such regression models, we modeled continuous predictors (including PCs) and the number of teeth with restricted cubic splines containing three knots, which allows for deviations from linearity [24, 29, 30]. Robust (heteroscedasticity consistent) variances of the HC3 type were computed as recommended [24, 31]. Joint tests were performed to evaluate the overall statistical effect of the selected major PCs on muscle thickness averaged across both sides. To evaluate the clinically relevant effect of an individual PC, the effect of the number of teeth was chosen as the reference effect, given that tooth number has been shown to influence overall facial shape and development [32].

For the baseline characteristics, the quartiles of continuous variables were associated with the two muscles.

## Results

### Principal component analysis

Different PCs captured distinct patterns of anatomical variation, which summed to 67.4% for the first seven PCs, each contributing at least 5% of the total variance. PC1 accounted for the largest proportion of variance (19.7%; Table 1) and primarily reflected variations in facial height and cranial base length.

**Table 1:** Baseline characteristics (SHIP-TREND-0, 2008–2012, *n* = 692)

	Category or range	*n*	Masseter muscle		Medial pterygoid muscle	
			Mean ± SD	Median(1st ; 3rd quartile)	Mean ± SD	Median(1st ; 3rd quartile)
			cm²	cm²	cm²	cm²
Sex	female	339	3.61 ± 0.78	3.54 (3.00; 4.14)	2.26 ± 0.61	2.17 (1.89; 2.58)
	male	353	4.76 ± 1.06	4.60 (4.01; 5.36)	2.86 ± 0.63	2.82 (2.46; 3.26)
Age, years	22–29	54	4.37 ± 0.97	4.26 (3.74; 4.83)	2.76 ± 0.71	2.73 (2.38; 3.12)
	30–39	70	4.49 ± 1.14	4.38 (3.90; 5.03)	2.70 ± 0.70	2.75 (2.16; 3.01)
	40–49	157	4.44 ± 1.13	4.28 (3.64; 5.04)	2.59 ± 0.63	2.57 (2.16; 2.97)
	50–59	181	4.21 ± 1.12	4.08 (3.46; 4.78)	2.61 ± 0.76	2.49 (2.11; 3.04)
	60–69	154	3.91 ± 0.99	3.75 (3.13; 4.62)	2.46 ± 0.67	2.46 (1.99; 2.86)
	70–81	76	3.86 ± 0.95	3.75 (3.34; 4.35)	2.38 ± 0.51	2.33 (2.14; 2.63)
Education,	< 10	95	3.84 ± 0.96	3.77 (3.04; 4.36)	2.38 ± 0.60	2.36 (1.95; 2.87)
years	10	352	4.16 ± 1.11	4.04 (3.31; 4.79)	2.54 ± 0.68	2.48 (2.08; 2.93)
	> 10	245	4.39 ± 1.07	4.32 (3.73; 4.98)	2.68 ± 0.71	2.65 (2.20; 3.08)
Teeth	0	20	3.57 ± 0.57	3.45 (3.14; 4.02)	2.27 ± 0.46	2.32 (1.95; 2.51)
	1–9	67	3.48 ± 0.83	3.43 (2.87; 3.96)	2.39 ± 0.66	2.41 (1.90; 2.85)
	10–19	100	3.85 ± 0.91	3.81 (3.14; 4.29)	2.39 ± 0.57	2.29 (2.05; 2.67)
	20–25	200	4.12 ± 1.08	3.94 (3.43; 4.78)	2.48 ± 0.70	2.39 (2.06; 2.91)
	26–28	197	4.43 ± 1.07	4.35 (3.72; 5.01)	2.69 ± 0.73	2.68 (2.18; 3.08)
	29–32	108	4.82 ± 1.09	4.72 (4.07; 5.45)	2.84 ± 0.59	2.82 (2.47; 3.14)
Height, cm	145–164	197	3.61 ± 0.85	3.52 (2.98; 4.11)	2.21 ± 0.62	2.15 (1.85; 2.49)
	165–171	173	3.95 ± 0.91	3.89 (3.25; 4.55)	2.44 ± 0.61	2.46 (2.05; 2.83)
	172–177	156	4.61 ± 1.09	4.45 (3.87; 5.31)	2.83 ± 0.59	2.78 (2.39; 3.26)
	178–194	166	4.77 ± 1.09	4.60 (4.07; 5.30)	2.87 ± 0.69	2.82 (2.46; 3.28)
Weight, kg	46.6–71.7	173	3.59 ± 0.84	3.50 (2.97; 4.15)	2.26 ± 0.61	2.18 (1.88; 2.62)
	71.8–81.0	174	4.17 ± 1.10	4.06 (3.44; 4.68)	2.50 ± 0.62	2.48 (2.08; 2.88)
	81.1–91.5	172	4.44 ± 1.06	4.38 (3.63; 5.03)	2.63 ± 0.65	2.64 (2.17; 2.96)
	91.6–121.5	173	4.60 ± 1.07	4.42 (3.89; 5.25)	2.88 ± 0.71	2.78 (2.42; 3.32)
Centroid size,	164–176	173	3.42 ± 0.74	3.33 (2.90; 3.92)	2.12 ± 0.58	2.11 (1.73; 2.46)
cm	176–183	173	3.90 ± 0.85	3.90 (3.25; 4.41)	2.40 ± 0.61	2.33 (2.07; 2.70)
	183–188	173	4.52 ± 0.98	4.38 (3.84; 5.04)	2.75 ± 0.54	2.73 (2.43; 3.09)
	188–203	173	4.96 ± 1.08	4.82 (4.14; 5.66)	3.00 ± 0.66	2.95 (2.54; 3.43)
PC1 (19.7%)	-0.066 – -0.016	173	4.69 ± 1.11	4.58 (3.92; 5.36)	2.88 ± 0.65	2.84 (2.46; 3.32)
	-0.016–0.000	173	4.34 ± 1.08	4.17 (3.54; 5.03)	2.61 ± 0.71	2.59 (2.11; 3.03)
	0.000–0.016	173	4.10 ± 1.03	4.01 (3.46; 4.60)	2.49 ± 0.61	2.45 (2.11; 2.82)
	0.016–0.110	173	3.67 ± 0.88	3.60 (3.00; 4.14)	2.28 ± 0.63	2.21 (1.90; 2.63)
PC2 (12.5%)	-0.056 – -0.015	173	4.07 ± 1.06	3.98 (3.38; 4.64)	2.49 ± 0.69	2.37 (2.08; 2.91)
	-0.015 – -0.001	173	4.26 ± 1.14	4.13 (3.42; 4.93)	2.59 ± 0.62	2.57 (2.15; 2.98)
	-0.001–0.014	173	4.33 ± 1.07	4.29 (3.59; 5.01)	2.60 ± 0.71	2.57 (2.08; 2.95)
	0.014–0.079	173	4.13 ± 1.09	3.98 (3.38; 4.73)	2.59 ± 0.72	2.55 (2.15; 2.94)
PC3 (9.2%)	-0.076 – -0.012	173	4.16 ± 1.08	4.08 (3.43; 4.78)	2.55 ± 0.70	2.47 (2.10; 2.88)
	-0.012–0.000	173	4.13 ± 1.04	4.00 (3.38; 4.75)	2.53 ± 0.68	2.48 (2.09; 2.92)
	0.000–0.012	173	4.22 ± 1.14	4.07 (3.38; 4.98)	2.57 ± 0.70	2.56 (2.11; 3.06)
	0.012–0.051	173	4.29 ± 1.11	4.19 (3.56; 4.84)	2.62 ± 0.67	2.61 (2.13; 3.01)
PC4 (7.3%)	-0.044 – -0.010	173	4.06 ± 1.06	3.93 (3.25; 4.73)	2.47 ± 0.68	2.44 (2.07; 2.88)
	-0.010–0.000	173	4.17 ± 1.18	3.97 (3.34; 4.77)	2.49 ± 0.66	2.46 (2.02; 2.85)
	0.000–0.010	173	4.31 ± 1.12	4.19 (3.46; 5.12)	2.59 ± 0.74	2.61 (2.06; 2.99)
	0.010–0.052	173	4.26 ± 0.99	4.14 (3.59; 4.80)	2.72 ± 0.64	2.70 (2.28; 3.09)
PC5 (6.6%)	-0.040 – -0.010	173	4.10 ± 1.12	3.93 (3.28; 4.69)	2.53 ± 0.68	2.43 (2.08; 2.95)
	-0.010–0.000	173	4.14 ± 1.04	4.02 (3.40; 4.80)	2.51 ± 0.70	2.48 (2.02; 2.85)
	0.000–0.010	173	4.36 ± 1.16	4.19 (3.42; 5.04)	2.61 ± 0.69	2.61 (2.10; 3.06)
	0.010–0.055	173	4.19 ± 1.04	4.06 (3.59; 4.74)	2.62 ± 0.67	2.57 (2.15; 2.95)
PC6 (6.2%)	-0.044 – -0.010	173	4.26 ± 1.12	4.18 (3.52; 4.89)	2.63 ± 0.73	2.61 (2.10; 3.01)
	-0.010–0.000	173	4.19 ± 1.01	4.06 (3.46; 4.74)	2.50 ± 0.65	2.49 (2.11; 2.94)
	0.000–0.010	173	4.23 ± 1.21	4.13 (3.34; 4.82)	2.65 ± 0.69	2.59 (2.16; 2.95)
	0.010–0.045	173	4.12 ± 1.03	3.97 (3.41; 4.75)	2.49 ± 0.66	2.41 (1.99; 2.92)
PC7 (5.9%)	-0.041 – -0.010	173	4.21 ± 1.22	4.08 (3.30; 4.93)	2.64 ± 0.69	2.59 (2.17; 3.00)
	-0.010 – -0.001	173	4.14 ± 1.04	3.97 (3.43; 4.70)	2.51 ± 0.70	2.46 (1.98; 2.96)
	-0.001–0.010	173	4.12 ± 0.95	4.10 (3.46; 4.60)	2.54 ± 0.65	2.51 (2.08; 2.88)
	0.010–0.049	173	4.32 ± 1.15	4.15 (3.54; 5.02)	2.58 ± 0.70	2.56 (2.14; 3.01)
Overall		692	4.20 ± 1.09	4.08 (3.43; 4.80)	2.57 ± 0.69	2.52 (2.11; 2.95)

*PC* denotes principal component

For PCs, the proportion of anatomical variance resulting from the principal component analysis described in the Materials and Methods section and reported in the Results section is provided in parentheses

*PC1*: Facial height, cranial base length, and maxillary orientation (vertical vs. horizontal)

*PC2*: Maxillary width and facial prominence

*PC3*: Craniofacial asymmetry

*PC4*: Anteroposterior facial dimensions and posterior facial height

*PC5*–*PC7*: Subtle craniofacial shape variations and minor asymmetries

The categories of continuous variables, including PCs but excluding age, correspond to the four quartile groups (minimum – first quartile, first quartile plus a small unit – second quartile, second quartile plus a small unit – third quartile, third quartile plus a small unit – maximum; for centroid size and PCs, the left limits and maximum lie within the interval). For age, ten-year categories were designated

**Fig. 3:**
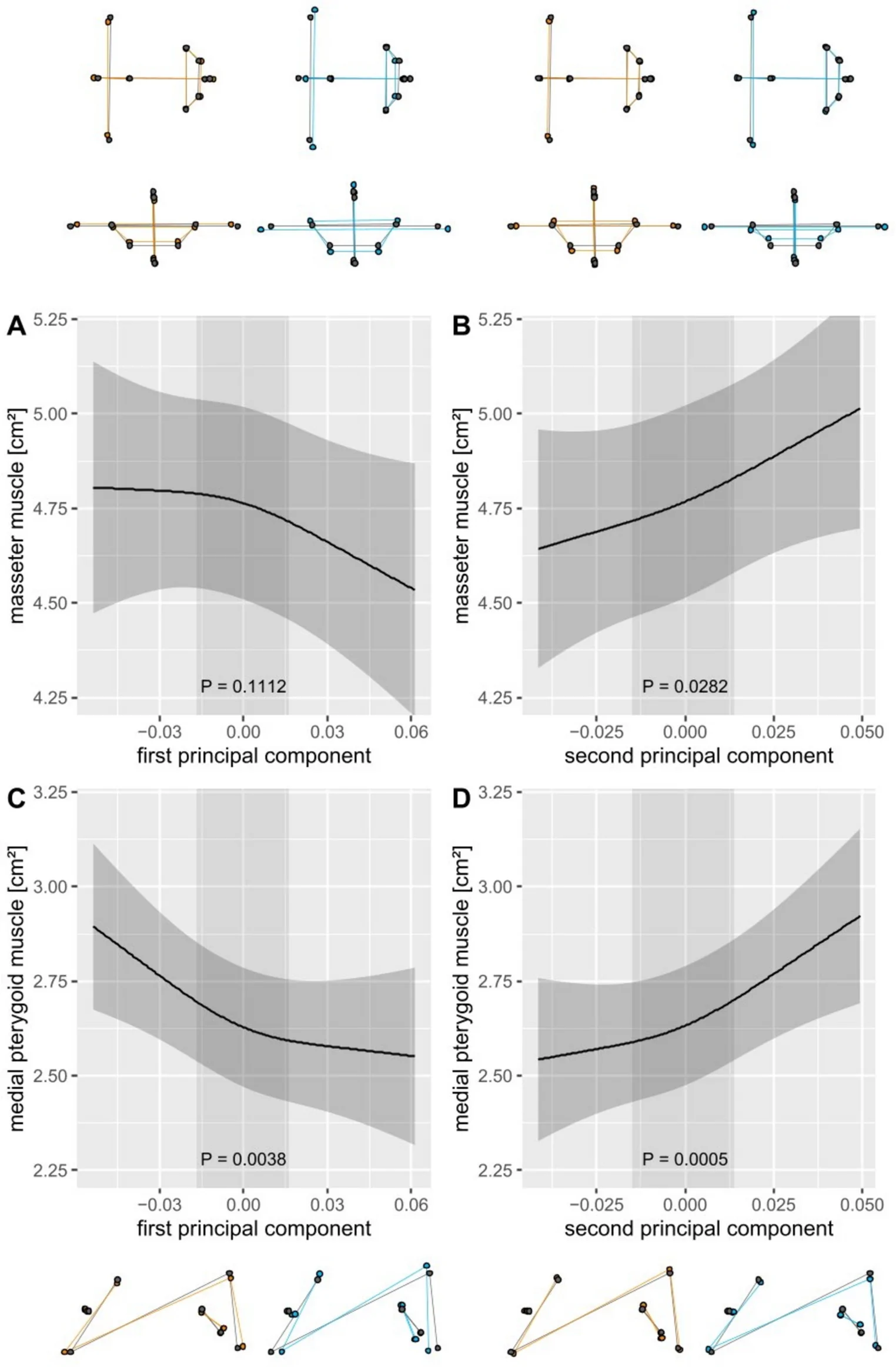
Association between the first or second principal component of anatomic variation and the masseter or medial pterygoid muscle, adjusted for the other principal components, sex, age, the interaction between sex and age, number of teeth, centroid,, centroid size, body weight, body height, and education. Dark grey: 95% confidence intervals. The vertical grey area represents the interquartile range of the principal component (first to third quartile). The shapes of the principal components are represented by point-based visualization. The craniofacial change in shape is shown in the transverse plane in the first visualization, in the frontal plane in the second and in the sagittal plane in the lowest visualization. MAG = 1

**Fig. 4:**
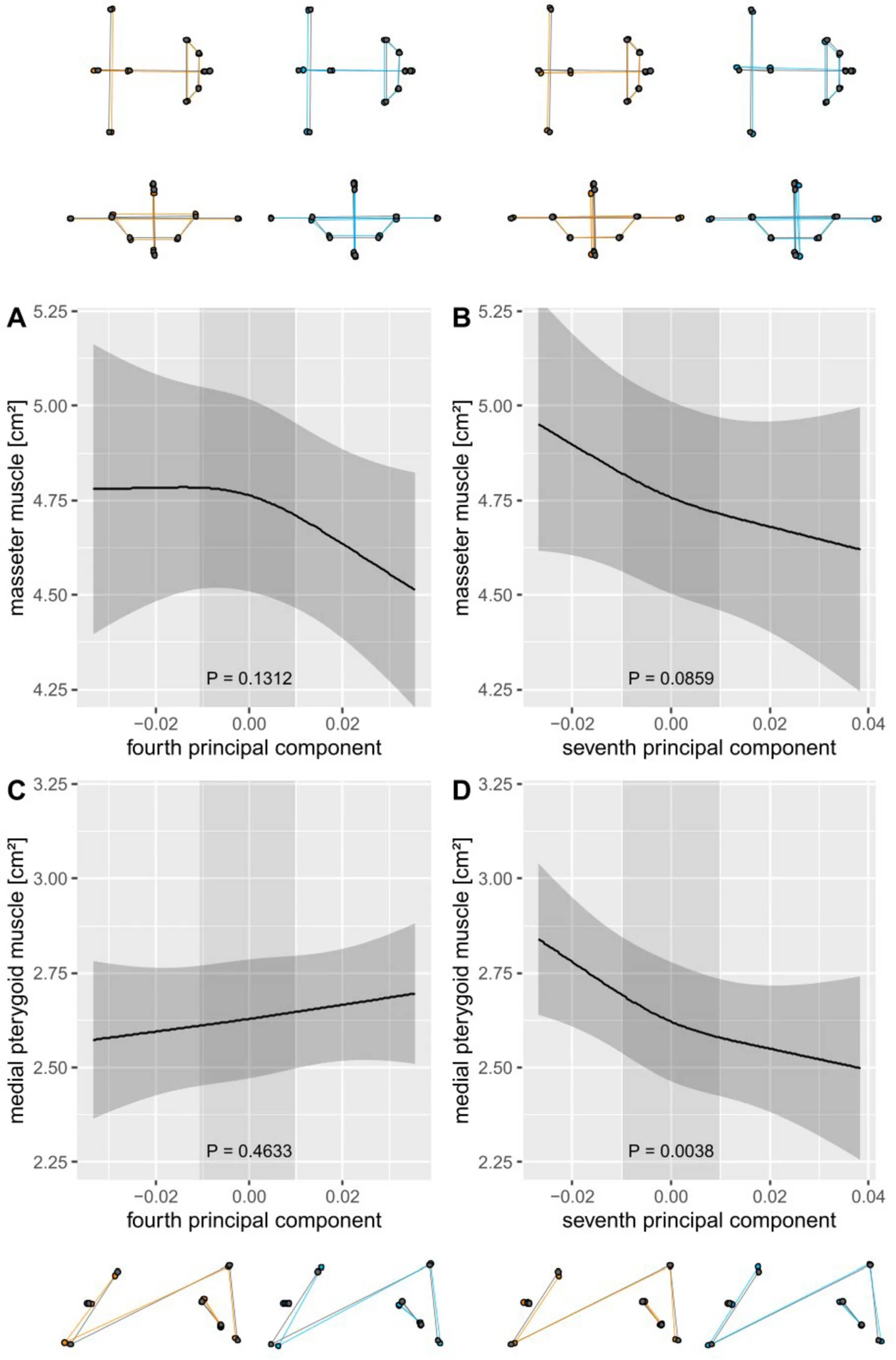
Association between the fourth or seventh principal component of anatomic variation and the masseter or medial pterygoid muscle, adjusted for the other principal components, sex, age, the interaction between sex and age, number of teeth, centroid size, body weight, body height, and education. Dark grey: 95% confidence intervals. The vertical grey area represents the interquartile range of the principal component (first to third quartile). The shapes of the principal components are represented by point-based visualization. The view of the visualization corresponds to those in Fig. 3. MAG = 1

These interpretations were derived from the comparison of the shape configurations in Figs. 3 and 4 representing the minimum (orange) and maximum (blue) along each principal component. In the transverse and frontal plane (first and second row), higher PC1 values were associated with facial elongation, a broader midface, whereas lower PC1 values indicated a shorter, more compact face. Additionally, individuals with high PC1 values exhibited a more vertical maxillary orientation and a longer cranial base, whereas those with low PC1 values showed a more horizontal maxillary orientation and a shorter cranial base (bottom row). These findings demonstrate that PC1 is associated with both facial shape and maxillary orientation (Fig. 3).

PC2 captured another major axis of variation, primarily associated with maxillary width and facial prominence. This interpretation was mainly based on the frontal and sagittal plane (second and bottom row), where the comparison of the minimum and maximum shape configurations showed a clear widening of the maxilla and midface at higher PC2 values, whereas lower PC2 values indicated a narrower maxilla. In the frontal plane (second row), higher PC2 values were also associated with slightly increased anterior facial height, although this effect was less pronounced than for PC1. In addition, the sagittal plane (bottom row) indicated greater nasal and midfacial prominence at higher PC2 values compared with lower PC2 values (Fig. 3).

The anatomical variations captured by PCs 3 through 7 were more nuanced. PC3 was associated with craniofacial asymmetries, lower values corresponded to reduced asymmetry. PC4 was associated with anterior–posterior facial dimensions. In the sagittal plane of the shape configurations (Fig. 4), higher PC4 values corresponded to a more prominent facial profile and increased posterior facial height, whereas lower values indicated a flatter profile. PCs 5, 6, and 7 represented even subtler morphological variations, potentially reflecting minor asymmetries and soft tissue variations.

### Baseline characteristics

The observed size of both the masseter and the pterygoid muscle differed considerably by sex, number of teeth, height, weight, centroid size, and PC1 (see Table 1). For the masseter muscle, the difference in size between the lower and upper quartile groups of PC1 was 1.02 cm² (4.69–3.67 in Table 1; the first quartile of PC1 was − 0.0016 and the third quartile was 0.0016). The observed difference in size between the first and third PC1 quartiles itself was 0.46 cm² (absolute interquartile range contrast in the “unadjusted” column of Table 2).

**Table 2:** Change in the coefficients of interest (PC1 – PC7) for the association with masseter and medial pterygoid muscle by including confounders (*n* = 692, using sampling weights derived from tooth count categories)

PC	Unadjusted	Adjusted for other PCs	Additionally adjusted for sex	Additionally adjusted for age and age by sex	Additionally adjusted for the number of teeth	Additionally adjusted for centroid size	Additionally adjusted for weight	Additionally adjusted for height	Additionally adjusted for education; final model	
	IQR contrast (95% CI), cm²	IQR contrast (95% CI), cm²	IQR contrast (95% CI), cm²	IQR contrast (95% CI), cm²	IQR contrast (95% CI), cm²	IQR contrast (95% CI), cm²	IQR contrast (95% CI), cm²	IQR contrast (95% CI), cm²	IQR contrast (95% CI), cm²	global *P* value
Masseter muscle										
1	-0.46 (-0.56 – -0.37)	-0.46 (-0.56 – -0.37)	-0.26 (-0.33 – -0.17)	-0.18 (-0.27 – -0.09)	-0.15 (-0.24 – -0.07)	-0.10 (-0.18 – -0.01)	-0.09 (-0.18–0.00)	-0.07 (-0.16–0.01)	-0.07 (-0.16–0.02)	0.1112
2	0.05 (-0.05–0.15)	0.04 (-0.05–0.13)	0.02 (-0.07–0.10)	0.08 (-0.01–0.17)	0.12 (0.04–0.21)	0.14 (0.06–0.22)	0.12 (0.04–0.21)	0.11 (0.02–0.19)	0.11 (0.03–0.20)	0.0282
3	0.05 (-0.05–0.16)	0.05 (-0.05–0.15)	0.04 (-0.05–0.13)	0.02 (-0.06–0.11)	0.02 (-0.06–0.11)	0.04 (-0.05–0.12)	0.03 (-0.05–0.11)	0.04 (-0.04–0.12)	0.04 (-0.04–0.12)	0.5096
4	0.09 (-0.01–0.19)	0.10 (0.00–0.20)	-0.12 (-0.22–0.02)	-0.09 (-0.20–0.01)	-0.10 (-0.19–0.00)	-0.08 (-0.17–0.02)	-0.09 (-0.18–0.01)	-0.06 (-0.16–0.03)	-0.07 (-0.17–0.02)	0.1312
5	0.11 (0.01–0.21)	0.13 (0.03–0.23)	-0.03 (-0.13–0.06)	-0.06 (-0.16–0.04)	-0.05 (-0.15–0.04)	-0.03 (-0.12–0.06)	-0.04 (-0.13–0.06)	-0.02 (-0.12–0.07)	-0.02 (-0.12–0.08)	0.6807
6	-0.02 (-0.14–0.09)	-0.04 (-0.14–0.07)	-0.02 (-0.11–0.07)	-0.03 (-0.12–0.06)	-0.02 (-0.10–0.07)	0.00 (-0.09–0.09)	-0.01 (-0.09–0.08)	0.01 (-0.08–0.10)	0.01 (-0.08–0.10)	0.9829
7	0.08 (-0.03–0.20)	0.07 (-0.04–0.19)	-0.06 (-0.17–0.05)	-0.12 (-0.22 – -0.01)	-0.13 (-0.23 – -0.04)	-0.11 (-0.21 – -0.02)	-0.12 (-0.22 – -0.03)	-0.11 (-0.20 – -0.02)	-0.11 (-0.20 – -0.01)	0.0859
Medial pterygoid muscle										
1	-0.30 (-0.36 – -0.24)	-0.30 (-0.36 – -0.24)	-0.21 (-0.27 – -0.15)	-0.17 (-0.23 – -0.11)	-0.16 (-0.22 – -0.10)	-0.11 (-0.18 – -0.05)	-0.11 (-0.17 – -0.04)	-0.10 (-0.16 – -0.04)	-0.10 (-0.17 – -0.04)	0.0038
2	0.08 (0.02–0.15)	0.08 (0.01–0.14)	0.06 (0.00–0.13)	0.11 (0.04–0.17)	0.12 (0.06–0.19)	0.14 (0.07–0.20)	0.12 (0.06–0.18)	0.11 (0.05–0.17)	0.11 (0.05–0.18)	0.0005
3	0.04 (-0.02–0.11)	0.05 (-0.01–0.10)	0.04 (-0.02–0.09)	0.03 (-0.02–0.09)	0.03 (-0.02–0.09)	0.04 (-0.01–0.10)	0.04 (-0.01–0.09)	0.04 (-0.01–0.10)	0.04 (-0.01–0.09)	0.2382
4	0.09 (0.02–0.15)	0.10 (0.04–0.16)	0.00 (-0.06–0.06)	0.02 (-0.04–0.08)	0.02 (-0.04–0.08)	0.04 (-0.02–0.09)	0.03 (-0.03–0.08)	0.04 (-0.02–0.10)	0.04 (-0.02–0.09)	0.4633
5	0.06 (0.00–0.12)	0.07 (0.01–0.13)	-0.01 (-0.07–0.05)	-0.02 (-0.08–0.04)	-0.02 (-0.11–0.01)	0.00 (-0.06–0.06)	-0.01 (-0.06–0.05)	0.00 (-0.06–0.06)	0.00 (-0.06–0.06)	0.5163
6	-0.04 (-0.11–0.02)	-0.05 (-0.11–0.01)	-0.05 (-0.11–0.01)	-0.05 (-0.11–0.00)	-0.05 (-0.11–0.01)	-0.04 (-0.10–0.02)	-0.05 (-0.11–0.01)	-0.04 (-0.10–0.01)	-0.05 (-0.10–0.01)	0.1469
7	-0.03 (-0.10–0.05)	-0.03 (-0.10–0.04)	-0.09 (-0.16 – -0.02)	-0.12 (-0.19 – -0.05)	-0.12 (-0.19 – -0.06)	-0.11 (-0.17 – -0.04)	-0.12 (-0.19 – -0.05)	-0.11 (-0.18 – -0.05)	-0.11 (-0.18 – -0.04)	0.0038

*PC* denotes principal component, *IQR* denotes interquartile range. The IQR contrast is calculated as the difference in muscle size for the third PC quartile minus muscle size for the first PC quartile (quartiles of PCs are given in Table 1; the IQR of PCs is represented by the darker vertical gray area in Figs. 3 and 4); the global *P* value refers to the entire range (minimum to maxim)

### Regression analysis

The association between PC1 and the masseter muscle decreased in absolute value from − 0.46 cm² to -0.26 -0.18 and − 0.10 cm² when sex, age, and centroid size were included as major confounders (Table 2). We considered an absolute contrast in masseter muscle CSA of 0.16 cm² for two teeth, within the range of 24 to 32 teeth, to be clinically important (for fewer teeth, the contrast was smaller). However, because the fully adjusted association between the masseter muscle and PC1 was − 0.07, with a CI ranging from − 0.16 to 0.02 (*P* = 0.1112; Fig. 3A), a clinically relevant association can be nearly excluded. Similar results were obtained for PC2 (Fig. 3B), PC4 (Fig. 4A), and PC7 (Fig. 4B) (Table 2).

The association between PC1 and the medial pterygoid muscle decreased in absolute value from − 0.30 cm² to -0.21 -0.17 and − 0.11 cm² when sex, age, and centroid size were included as major confounders (Table 2). Here, we considered an absolute contrast in medial pterygoid muscle size of 0.07 cm² for two teeth, within the range of 24 to 32 teeth, to be clinically important (for fewer teeth, the contrast was smaller). Thus, the fully adjusted association between the medial pterygoid muscle and PC1 was statistically significant for the entire range (*P* = 0.0038; Table 2) and clinically relevant for the contrast of -0.10 cm² resulting from the area of 2.59 cm² at the third quantile of 0.016 for PC1 minus the area of 2.69 cm² at the first quantile of -0.016 for PC1 (Fig. 3C; Table 2). Clinically relevant and statistically significant results were also obtained for PC2 (Fig. 3D) and PC7 (Fig. 4D) (Table 2). Contrary to PC1, PC2 was only slightly confounded by sex, centroid size, and the other variables (Table 2). PC7, however, was confounded by sex to the extent that the contrast became clinically relevant as defined above (Table 2).

**Fig. 5:**
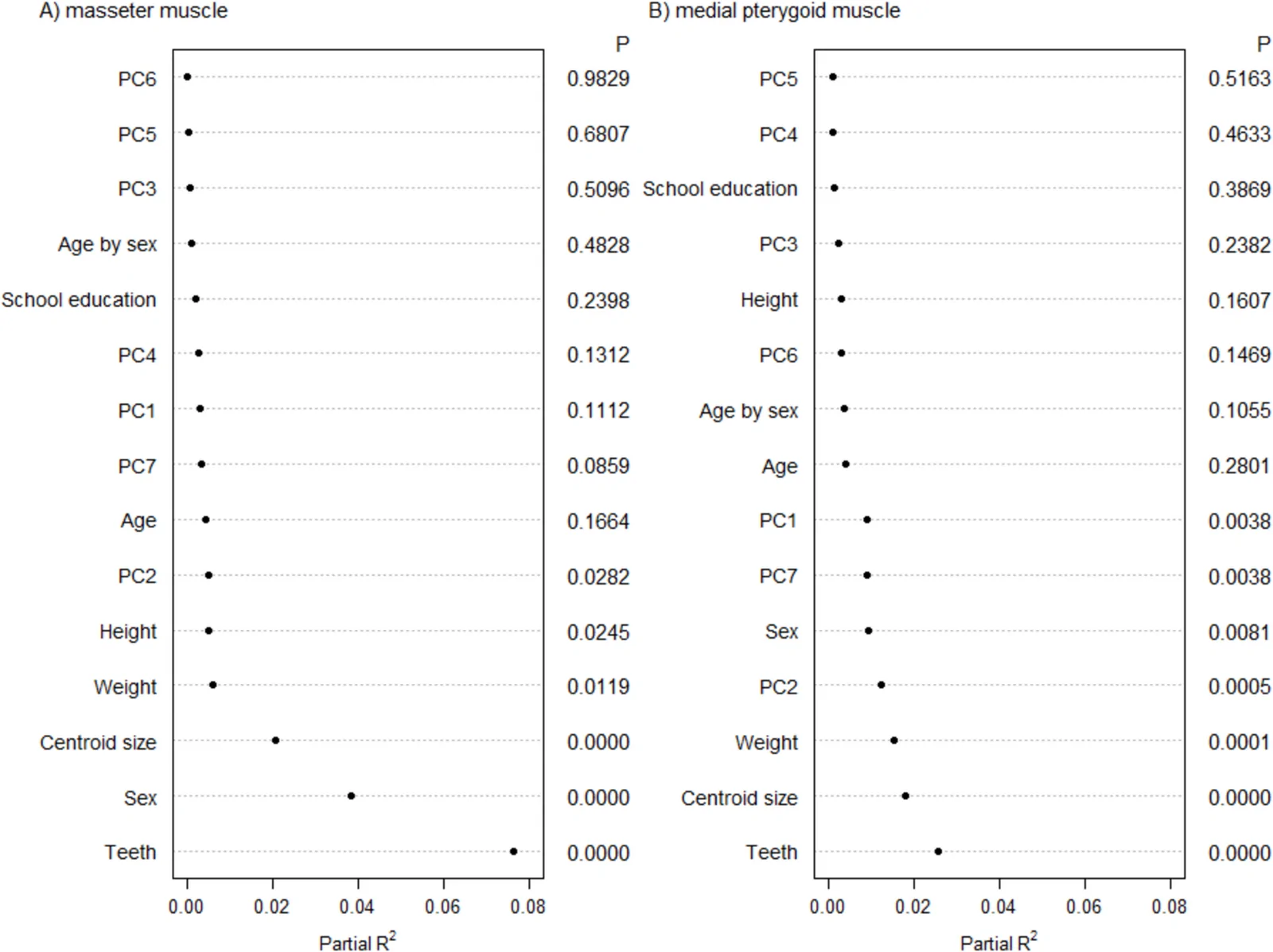
Partial R² (x-axis) and P value (right column) for the masseter and medial pterygoid muscle model’s single covariates

Comparing the two muscle models, the importance of PCs for craniofacial morphology was more pronounced for the pterygoid muscle than for the masseter muscle (Fig. 5) even though the adjusted R² was higher in the masseter muscle model (R²_adjusted_ = 0.348 and 0.450, respectively; joint test of the seven PCs: *P* < 0.0001 and *P* = 0.0225 in the pterygoid and masseter muscle models, respectively). The number of teeth, sex, and centroid size were important predictors in the masseter muscle model (partial R²: 0.076, 0.039, and 0.021, respectively; Fig. 5A), whereas PCs were clearly less important (largest partial R² = 0.005 for PC2; group partial R² = 0.016 for PC1 through PC7). In the pterygoid muscle model, however, PC1, PC2, and PC7 together with sex belonged to the set of second important predictors (partial R² = 0.009, 0.012, 0.009, and 0.010, respectively; Fig. 5B), here after the number of teeth, centroid size, and weight (partial R² = 0.026, 0.018, and 0.015, respectively). In contrast, the group partial R² for PC1 through PC7 was 0.038.

Regression models without sampling weights produce similar results (Supplementary Information, Table S2 and Figures S1, S2, and S3).

## Discussion

This study investigated the association between the masseter and the medial pterygoid muscles and craniofacial morphology using geometric morphometrics and regression analysis on data from a population-based study from east Germany. Our main finding was that the CSA of the medial pterygoid muscle was more associated with craniofacial variation, whereas the masseter muscle was primarily associated with functional factors such as dental status and sex. This finding aligns with the distinct functional roles of these muscles in mastication and jaw movement [8].

Facial morphology and functional factors such cannot be considered entirely independent. In our study, medial pterygoid CSA showed clearer associations with craniofacial shape variables, whereas masseter CSA was more strongly related to sex and dental status. This pattern is consistent with previous work suggesting that craniofacial morphology and masticatory musculature are linked through biomechanical loading, while also reflecting a multifactorial relationship [33–36]. One possible explanation is that the medial pterygoid may reflect craniofacial architecture more closely, whereas the masseter may be more strongly influenced by functional factors and dental status. Nonetheless, these findings cannot be interpreted causally in the present cross-sectional study.

Previous studies have pointed out that the anatomical characteristics of muscles, including their insertion zones and orientation, can partly explain functional differences between the masseter and medial pterygoid muscles. The masseter originates from the zygomatic arch, whereas the medial pterygoid muscle partly originates directly from the upper jaw [4].

The masseter plays a crucial role in mastication as the primary elevator of the mandible. Misalignment of teeth or changes in occlusion significantly alter load distribution on the masseter, affecting its development. Grünheid et al. [37] emphasized the masseter’s sensitivity to occlusal variables, demonstrating that unilateral mastication or malocclusions can lead to asymmetrical muscle development. Daboul et al. [8] further highlighted the medial pterygoid’s deeper positioning and diffuse attachment pattern, which reduce its direct dependence on occlusion forces compared to the masseter. Its role in lateral and rotational jaw movements renders it less reliant on vertical bite force.

In our study, PC1, which primarily reflects facial height and cranial base length, and PC2, which represents maxillary width and facial prominence, were the most meaningful axes of craniofacial variation. They were associated with the medial pterygoid muscle: longer faces were associated with thinner pterygoid muscles, and wider midfaces were associated with thicker pterygoid muscles. One possible explanation is that altered facial height modifies the medial pterygoid muscle’s mechanical advantage. A longer face may create a more efficient lever system, potentially leading to maladaptive reduced muscle development. In contrast, a shorter face may not have this mechanical advantage, which leads to increase loading on the muscle and further development.

Several studies have investigated the association between craniofacial morphology and masticatory muscle size/shape, yielding varied results. Soyoye et al. [38] reported an inverse association between the thickness of the masseter and long facial patterns, which contrasts with the current study’s finding of no strong influence of craniofacial morphology on the masseter [39]. Similarly, Chan et al. [10] and other studies reported positive associations between masseter and medial pterygoid muscle thickness and facial width [12, 39, 40], which aligns partially with the current study’s finding on the medial pterygoid. However, our study also found a negative association between medial pterygoid thickness and longer faces, a finding not explicitly addressed in previous literature. Nonetheless, the heterogeneity of populations and methods used across these studies makes definitive comparisons challenging, while inconsistencies in defining craniofacial types (e.g., long vs. short faces) further complicates interpretations.

The substantial association between sex and muscle thickness is consistent with previous research. Several studies have reported that males typically exhibit thicker masticatory muscles than females. These differences are commonly attributed to variations in muscle mass, hormonal influences, and overall craniofacial morphology [41].

The association between PC7 and the pterygoid muscle, which may reflect subtle craniofacial asymmetries, merits a discussion. Research by Ferrario et al. [7] suggests that even minor asymmetries can influence muscle function and development, particularly in muscles responsible for complex movements, such as the medial pterygoid. Eo et al. [42] also found that the degree of facial asymmetry increases with muscle volume asymmetry. The extended side exhibited a more substantial reduction in masticatory muscle volume relative to the shortened side. Our study has several limitations. Geometric morphometric analysis, while powerful, is sensitive to the quality and consistency of the imaging data. Small errors in landmark placement or variations in image resolution can introduce noise into the analysis. Additionally, the CSA measurements of the masticatory muscles can be influenced by factors such as hydration status, muscle activity level at the time of imaging, and individual variability in muscle composition. Daboul et al. [8] highlighted similar challenges, noting that factors like muscle tonicity and imaging angle can also affect measurement accuracy. Moreover, the cross-sectional design of the study limits the ability to draw causal inferences about the associations observed. Future studies could address these issues by employing longitudinal designs and incorporating additional factors such as lifestyle or dietary habits. Variations in masticatory function, such as differences in diet consistency or muscle loading, have been shown to impact both muscle morphology and craniofacial structure [43, 44]. Increased masticatory activity is often associated with thicker masseter muscles and broader facial morphology.

Notwithstanding these limitations, several strengths of our study merit consideration. Procrustes analysis, which can be considered complementary to, rather than competing with conventional analysis, allows for the investigation of new aspects, such as shape, rather than just the distances between landmarks. To address the most important mathematical assumptions in regression analysis, we modeled departures from linearity [24, 29, 30]. Importantly, our MRI study had a large sample size. As the study population consisted exclusively of German residents from West Pomerania, our findings may be limited in their generalizability to more genetically and socio-environmentally diverse populations. On the other hand, the relatively homogeneous population structure may reduce confounding factors and allow clearer detection of associations between craniofacial morphology and masticatory musculature.

In summary, the associations identified between the medial pterygoid muscle and facial types suggest an anatomical basis that could be relevant to the study of myofunctional disorders. Further research, however, is needed to determine its clinical significance.

## Supplementary Information

Below is the link to the electronic supplementary material.

Supplementary Material 1

## Data Availability

The data that supports the findings of this study are available from Forschungsverbund Community Medicine. Restrictions apply to the availability of these data, which were used under license for this study. Data are available from https://fvcm.med.uni-greifswald.de/dd_service/data_use_intro.php with the permission of Forschungsverbund Community Medicine.
