# Supplementary material for: Craniofacial morphology and its association with masseter and medial pterygoid muscle thickness among adults from a population-based sample
Source: Clin Oral Investig. 2026 Aug 25;30(9):411. doi: 10.1007/s00784-026-07102-4 (PMC13506625; doi:10.1007/s00784-026-07102-4)
Supplement: Supplementary file 1 [file 784_2026_7102_MOESM1_ESM.docx]

Supplementary Information

Clinical Oral Investigations

Craniofacial morphology and its association with masseter and medial pterygoid muscle thickness among adults from a population-based sample

Annalena Spilz^1^, Christian Schwahn ^1^, Karl-Friedrich Krey², Robin Bülow³, Henry Völzke⁴, Markus Krüger^1^ and Amro Daboul^1^

Affiliations

^1^ Department of Prosthetic Dentistry, Gerodontology, and Biomaterials, University Medicine Greifswald, Fleischmannstr. 42-44, 17475 Greifswald, Germany

² Department of Orthodontics, University Medicine Greifswald, Fleischmannstraße 42-44, 17475 Greifswald, Germany

³ Institute of Diagnostic Radiology and Neuroradiology, University Medicine Greifswald, 17475 Greifswald, Germany

⁴ Institute for Community Medicine, University Medicine Greifswald, Walther Rathenau Str. 48, 17475 Greifswald, Germany

Email addresses

annalena_spilz@gmx.de, christian.schwahn@med.uni-greifswald.de, karl-friedrich.krey@uni-greifswald.de, [robin.buelow@med.uni-greifswald.de](mailto:robin.buelow@med.uni-greifswald.de), voelzke@uni-greifswald.de, [markuskr@uni-greifswald.de](mailto:markuskr@uni-greifswald.de), daboula@uni-greifswald.de

Corresponding author

Annalena Spilz, Zentrum für Zahn-, Mund- und Kieferheilkunde, Walther-Rathenau-Straße 42a, 17475 Greifswald, [annalena_spilz@gmx.de](mailto:annalena_spilz@gmx.de)

## Regression Analysis (not using inverse probability weights)

The association between PC1 and the masseter muscle decreased in absolute value from ‑0.46 cm² to -0.24, -0.17 and -0.11 cm² when sex, age, and centroid size were included as major confounders (Table S2). We considered an absolute contrast in masseter muscle CSA of 0.16 cm² for two teeth, within the range of 24 to 32 teeth, to be clinically important (for fewer teeth, the contrast was smaller). However, because the fully adjusted association between the masseter muscle and PC1 was -0.09, with a CI ranging from -0,17 to 0.00 (P = 0.0759; Figure S1A), a clinically relevant association can be nearly excluded. Similar results were obtained for PC2 (Figure S1B), PC4 (Figure S2A), and PC7 (Figure S2B) (Table S2).

The association between PC1 and the medial pterygoid muscle decreased in absolute value from ‑0.29 cm² to -0.19, -0.16 and -0.11 cm² when sex, age, and centroid size were included as major confounders (Table S2). Here, we considered an absolute contrast in medial pterygoid muscle size of 0.07 cm² for two teeth, within the range of 24 to 32 teeth, to be clinically important (for fewer teeth, the contrast was smaller). Thus, the fully adjusted association between the medial pterygoid muscle and PC1 was statistically significant for the entire range (P = 0.0034; Table 2) and clinically relevant for the contrast of -0.10 cm² resulting from the area of 2.61 cm² at the third quantile of 0.016 for PC1 minus the area of 2.71 cm² at the first quantile of -0.016 for PC1 (Figure S1C; Table S2). Clinically relevant and statistically significant results were also obtained for PC2 (Figure S1D) and PC7 (Figure S2D) (Table 2). Contrary to PC1, PC2 was only slightly confounded by sex, centroid size, and the other variables (Table S2). PC7, however, was confounded by sex to the extent that the contrast became clinically relevant as defined above (Table S2).

Comparing the two muscle models, the importance of PCs for craniofacial morphology was more pronounced for the pterygoid muscle than for the masseter muscle (Figure S3) even though the adjusted R² was higher in the masseter muscle model (R²_adjusted_ = 0.332 and 0.441, respectively; joint test of the seven PCs: P < 0.0001 and P = 0.0313 in the pterygoid and masseter muscle models, respectively). The number of teeth, sex, centroid size, and weight were important predictors in the masseter muscle model (partial R²: 0.076, 0.037, 0.023, and 0.013, respectively; Figure S3A), whereas PCs were clearly less important (largest partial R² = 0.004 for PC4 and PC1; group partial R² = 0.016 for PC1 through PC7). In the pterygoid muscle model, however, PC1, PC2, and PC7 together with sex belonged to the set of second important predictors (partial R² = 0.010, 0.009, 0.009, and 0.009, respectively; Figure S3B), here after centroid size, the number of teeth, and weight (partial R² = 0.021, 0.020, and 0.015, respectively). In contrast, the group partial R² for PC1 through PC7 was 0.034.

**Figure S1.** Association between the first or second principal component of anatomic variation and the masseter or medial pterygoid muscle, adjusted for the other principal components, sex, age, the interaction between sex and age, number of teeth, centroid, , centroid size, body weight, body height, and education. Dark grey: 95% confidence intervals. The vertical grey area represents the interquartile range of the principal component (first to third quartile). The shapes of the principal components are represented by transformation grids. The craniofacial change in shape is shown in the transverse plane in the first grid, in the frontal plane in the second and in the sagittal plane in the lowest grid. MAG=1

**Figure S2.** Association between the fourth or seventh principal component of anatomic variation and the masseter or medial pterygoid muscle, adjusted for the other principal components, sex, age, the interaction between sex and age, number of teeth, centroid size, body weight, body height, and education. Dark grey: 95% confidence intervals. The vertical grey area represents the interquartile range of the principal component (first to third quartile). The shapes of the principal components are represented by transformation grids. The view of the grids corresponds to those in Figure 3. MAG=1

***Figure S3.*** *Partial R² (x-axis) and P value (right column) for the masseter and medial pterygoid muscle model's single covariates.*

Tables

***Table S1.*** *Baseline characteristics (SHIP-TREND-0, 2008 - 2012, n = 4420)*

|  |  | Included (n = 692) | |  | Excluded (n = 3726) | |  | Total (n = 4420) | |  |  |
| --- | --- | --- | --- | --- | --- | --- | --- | --- | --- | --- | --- |
|  | Category or range | n | % (column) |  | n | % (column) |  | n | % (column) |  | P value |
| Sex | female | 339 | 49.0 |  | 1936 | 51.9 |  | 2275 | 51.5 |  | 0.155 |
|  | male | 353 | 51.0 |  | 1792 | 48.1 |  | 2145 | 48.5 |  |  |
| Age, years | 22 – 29 | 54 | 7.8 |  | 330 | 8.9 |  | 384 | 8.7 |  | <0.001 |
|  | 30 – 39 | 70 | 10.1 |  | 627 | 16.8 |  | 697 | 15.8 |  |  |
|  | 40 – 49 | 157 | 22.7 |  | 722 | 19.4 |  | 879 | 19.9 |  |  |
|  | 50 – 59 | 181 | 26.2 |  | 750 | 20.1 |  | 931 | 21.1 |  |  |
|  | 60 – 69 | 154 | 22.3 |  | 689 | 18.5 |  | 843 | 19.1 |  |  |
|  | 70 – 81 | 76 | 11.0 |  | 610 | 16.4 |  | 686 | 15.5 |  |  |
| Education, | <10 | 95 | 13.7 |  | 913 | 24.7 |  | 1008 | 23.0 |  | <0.001 |
| years | 10 | 352 | 50.9 |  | 1913 | 51.9 |  | 2265 | 51.7 |  |  |
|  | >10 | 245 | 35.4 |  | 863 | 23.4 |  | 1108 | 25.3 |  |  |
| Teeth | 0 | 20 | 2.9 |  | 698 | 18.7 |  | 718 | 16.2 |  | <0.001 |
|  | 1 – 9 | 67 | 9.7 |  | 303 | 8.1 |  | 370 | 8.4 |  |  |
|  | 10 – 19 | 100 | 14.5 |  | 490 | 13.1 |  | 590 | 13.3 |  |  |
|  | 20 – 25 | 200 | 28.9 |  | 755 | 20.3 |  | 955 | 21.6 |  |  |
|  | 26 – 28 | 197 | 28.5 |  | 931 | 25.0 |  | 1128 | 25.5 |  |  |
|  | 29 – 32 | 108 | 15.6 |  | 551 | 14.8 |  | 659 | 14.9 |  |  |
| Height, cm | 137 – 164 | 197 | 28.5 |  | 1150 | 30.9 |  | 1347 | 30.5 |  | 0.206 |
|  | 165 – 171 | 173 | 25.0 |  | 997 | 26.8 |  | 1170 | 26.5 |  |  |
|  | 172 – 177 | 156 | 22.5 |  | 737 | 19.8 |  | 893 | 20.2 |  |  |
|  | 178 – 203 | 166 | 24.0 |  | 837 | 22.5 |  | 1003 | 22.7 |  |  |
| Weight, kg | 43.6 – 71.7 | 173 | 25.0 |  | 1185 | 32.5 |  | 1358 | 31.3 |  | 0.001 |
|  | 71.8 – 81.0 | 174 | 25.1 |  | 829 | 22.8 |  | 1003 | 23.1 |  |  |
|  | 81.1 – 91.5 | 172 | 24.9 |  | 748 | 20.5 |  | 920 | 21.2 |  |  |
|  | 91.6 – 169.2 | 173 | 25.0 |  | 880 | 24.2 |  | 1053 | 24.3 |  |  |

The P value is from the Pearson Chi-square test for the related crosstabulation.

***Table S2.*** *Change in the coefficients of interest (PC1 – PC7) for the association with masseter and medial pterygoid muscle by including confounders (n = 692)*

| PC | unadjusted | adjusted for other PCs | additionally adjusted for sex | additionally adjusted for age and age by sex | additionally adjusted for the number of teeth | additionally adjusted for centroid size | additionally adjusted for weight | additionally adjusted for height |  | additionally adjusted for education;  final model | |
| --- | --- | --- | --- | --- | --- | --- | --- | --- | --- | --- | --- |
|  | IQR contrast (95% CI), cm² | IQR contrast (95% CI), cm² | IQR contrast (95% CI), cm² | IQR contrast (95% CI), cm² | IQR contrast (95% CI), cm² | IQR contrast (95% CI), cm² | IQR contrast (95% CI), cm² | IQR contrast (95% CI), cm² |  | IQR contrast (95% CI), cm² | global  P value |
| **Masseter muscle** | | | | | | | | | | | |
| 1 | -0.46 (-0.55 – -0.36) | -0.45 (-0.54 – -0.35) | -0.24 (-0.33 – -0.14) | -0.17 (-0.27 – -0.08) | -0.17 (-0.25 – -0.08) | -0.11 (-0.20 – -0.02) | -0.10 (-0.19 – -0.01) | -0.09 (-0.17 – 0.00) |  | -0.0^,,^9 (-0.17 – 0.00) | 0.0759 |
| 2 | 0.07 (-0.04 – 0.18) | 0.07 (-0.04 – 0.17) | 0.06 (-0.03 – 0.14) | 0.10 (0.01 – 0.19) | 0.12 (0.03 – 0.21) | 0.14 (0.05 – 0.23) | 0.10 (0.01 – 0.19) | 0.09 (-0.01 – 0.18) |  | 0.09 (0.00 – 0.19) | 0.1337 |
| 3 | 0.05 (-0.06 – 0.17) | 0.05 (-0.06 – 0.16) | 0.04 (-0.06 – 0.14) | 0.03 (-0.07 – 0.12) | 0.04 (-0.06 – 0.13) | 0.06 (-0.04 – 0.15) | 0.05 (-0.04 – 0.15) | 0.07 (-0.03 – 0.16) |  | 0.06 (-0.03 – 0.16) | 0.3464 |
| 4 | 0.11 (0.00 – 0.22) | 0.10 (0.00 – 0.20) | -0.12 (-0.23 – 0.02) | -0.11 (-0.22 – 0.00) | -0.12 (-0.23 – -0.02) | -0.10 (-0.20 – 0.00) | -0.12 (-0.22 – -0.02) | -0.10 (-0.20 – 0.01) |  | -0.10 (-0.20 – 0.00) | 0.0698 |
| 5 | 0.10 (-0.01 – 0.20) | 0.11 (0.00 – 0.21) | -0.03 (-0.13 – 0.07) | -0.04 (-0.14 – 0.06) | -0.04 (-0.14 – 0.05) | -0.02 (-0.11 – 0.07) | -0.03 (-0.12 – 0.07) | -0.02 (-0.11 – 0.08) |  | -0.02 (-0.11 – 0.08) | 0.5876 |
| 6 | -0.04 (-0.15 – 0.07) | -0.04 (-0.14 – 0.07) | 0.01 (-0.08 – 0.10) | 0.01 (-0.08 – 0.10) | 0.02 (-0.07 – 0.11) | 0.02 (-0.07 – 0.11) | 0.02 (-0.07 – 0.10) | 0.03 (-0.05 – 0.12) |  | 0.03 (-0.06 – 0.12) | 0.7523 |
| 7 | 0.09 (-0.03 – 0.20) | 0.09 (-0.02 – 0.20) | -0.04 (-0.14 – 0.07) | -0.08 (-0.19 – 0.02) | -0.10 (-0.20 – 0.00) | -0.08 (-0.17 – 0.01) | -0.09 (-0.19 – 0.00) | -0.08 (-0.17 – 0.01) |  | -0.08 (-0.17 - 0.02) | 0.2274 |
| **Medial pterygoid muscle** | | | | | | | | | | | |
| 1 | -0.29 (-0.35 – -0.23) | -0.29 (-0.35 – -0.23) | -0.19 (-0.25 – -0.13) | -0.16 (-0.22 – -0.10) | -0.16 (-0.22 – -0.09) | -0.11 (-0.18 – -0.05) | -0.11 (-0.17 – -0.04) | -0.10 (-0.17 – -0.04) |  | -0.10 (-0.17 – -0.04) | 0.0034 |
| 2 | 0.07 (0.00 – 0.15) | 0.07 (0.00 – 0.14) | 0.07 (0.00 – 0.14) | 0.11 (0.04 – 0.17) | 0.11 (0.04 – 0.18) | 0.12 (0.06 – 0.19) | 0.11 (0.04 – 0.17) | 0.10 (0.03 – 0.17) |  | 0.10 (0.04 – 0.17) | 0.0067 |
| 3 | 0.05 (-0.02 – 0.12) | 0.05 (-0.02 – 0.11) | 0.04 (-0.02 – 0.11) | 0.04 (-0.02 – 0.10) | 0.04 (-0.02 – 0.10) | 0.06 (0.00 – 0.12) | 0.06 (0.00 – 0.12) | 0.06 (0.00 – 0.12) |  | 0.06 (0.00 – 0.11) | 0.1838 |
| 4 | 0.11 (0.04 – 0.18) | 0.11 (0.04 – 0.17) | 0.00 (-0.06 – 0.06) | 0.02 (-0.05 – 0.08) | 0.01 (-0.05 – 0.08) | 0.03 (-0.03 – 0.09) | 0.02 (-0.04 – 0.08) | 0.03 (-0.03 – 0.09) |  | 0.03 (-0.03 – 0.09) | 0.5708 |
| 5 | 0.06 (-0.01 – 0.12) | 0.06 (-0.01 – 0.12) | -0.01 (-0.07 – 0.05) | -0.01 (-0.07 – 0.05) | -0.01 (-0.07 – 0.05) | 0.00 (-0.05 – 0.06) | 0.00 (-0.06 – 0.06) | 0.00 (-0.06 – 0.06) |  | 0.00 (-0.06 – 0.07) | 0.5655 |
| 6 | -0.04 (-0.11 – 0.02) | -0.05 (-0.11 – 0.01) | -0.03 (-0.08 – 0.03) | -0.03 (-0.09 – 0.03) | -0.03 (-0.08 – 0.03) | -0.03 (-0.08 – 0.03) | -0.03 (-0.09 – 0.02) | -0.03 (-0.09 – 0.03) |  | -0.03 (-0.09 – 0.03) | 0.4757 |
| 7 | -0.03 (-0.10 – 0.04) | -0.03 (-0.11 – 0.04) | -0.10 (-0.17 – -0.02) | -0.11 (-0.19 – -0.04) | -0.12 (-0.19 – -0.05) | -0.10 (-0.17 – -0.04) | -0.12 (-0.19 – -0.05) | -0.11 (-0.18 – -0.04) |  | -0.11 (-0.18 – -0.04) | 0.0061 |

*PC denotes principal component. IQR denotes interquartile range. The IQR contrast is calculated as the difference in muscle size for the third PC quartile minus muscle size for the first PC quartile (quartiles of PCs are given in Table 1; the IQR of PCs is represented by the darker vertical gray area in Figures 3 and 4); the global P value refers to the entire range (minimum to maxim)*
